# Pressure Control Ventilation Versus Volume Control Ventilation in Laparoscopic Surgery: A Narrative Review

**DOI:** 10.7759/cureus.66916

**Published:** 2024-08-15

**Authors:** Urvi Sawant, Jayshree Sen, Sheetal Madavi

**Affiliations:** 1 Anesthesiology, Jawaharlal Nehru Medical College, Datta Meghe Institute of Higher Education and Research, Wardha, IND

**Keywords:** plateau pressure, peak airway pressure, volume control ventilation, pressure control ventilation, aroscopic surgery

## Abstract

This review compares the safety and effectiveness of volume control ventilation (VCV) and pressure control ventilation (PCV) during laparoscopic surgery. Nine studies were chosen for in-depth examination following the application of stringent inclusion and exclusion criteria to the 184 publications that the literature search turned up. PCV is well-known for its capacity to preserve lower peak airway pressures during laparoscopic procedures, lowering the risk of volutrauma and barotrauma and enhancing oxygenation under these conditions of elevated intra-abdominal pressures. On the other hand, VCV guarantees a constant tidal volume and offers accurate ventilation management, both of which are essential for preserving stable carbon dioxide levels. VCV, however, may result in higher peak airway pressures, raising the risk of lung damage brought on by a ventilator. Research indicates that PCV provides better respiratory mechanics management during laparoscopic surgery, but VCV consistent tidal volume delivery is useful in some clinical situations. When choosing between PCV and VCV, the anesthesia team's experience, the demands of each patient, and the surgical circumstances should all be taken into consideration. Real-time monitoring tools and sophisticated ventilatory technology are essential for maximizing ventilation techniques. Further improving patient outcomes can be achieved by incorporating multimodal anesthesia approaches, such as the use of muscle relaxants and customized intraoperative fluid management. Muscle relaxants optimize conditions for mechanical ventilation by ensuring adequate muscle relaxation, reducing the risk of ventilator-associated lung injury, and enabling more precise control of ventilation parameters. Tailored intraoperative fluid management helps maintain optimal lung mechanics by avoiding fluid overload, which can lead to pulmonary edema and compromised gas exchange, necessitating adjustments in ventilation strategy. While both ventilation modalities can be utilized efficiently, the research suggests that PCV may be more advantageous in controlling oxygenation and airway pressures. In the dynamic and demanding world of laparoscopic surgery, ongoing research and clinical innovation are crucial to improving these tactics and guaranteeing the best possible treatment. In order to obtain the best possible patient outcomes during laparoscopic surgeries, this review emphasizes the significance of customized breathing techniques.

## Introduction and background

Pressure control ventilation (PCV) and volume control ventilation (VCV) are two major modes of mechanical ventilation used for laparoscopic surgeries, each differing in their physiological and clinical implications [1]. The decision between these two strategies is important because it can considerably change the results of patients, especially in laparoscopic scenarios where the intra-abdominal pressures are high. This elevation has an impact on the mechanics of respiration coupled with hemodynamics secondary to it; thus, optimal ventilation strategy becomes a cornerstone during anesthetic care [2]. PCV is characterized by the delivery of a set of inspiratory pressures over a predetermined time, allowing for variations in tidal volume based on changes in lung compliance and resistance [3]. This mode is particularly advantageous in laparoscopic surgery due to its potential to provide better oxygenation and lower peak airway pressures, reducing the risk of barotraumas and volume trauma. Studies have suggested that PCV can be beneficial in maintaining stable respiratory mechanics despite the increased intra-abdominal pressures common in laparoscopic procedures [4].

VCV, on the other hand, guarantees constant minute ventilation by delivering a predetermined tidal volume with each breath [5]. The ability to precisely regulate carbon dioxide levels and ensure proper breathing in spite of the changing respiratory dynamics that arise during laparoscopic surgery can be a benefit of this modality. However, especially in patients with decreased lung compliance, the fixed tidal volume may result in higher peak airway pressures and thereby raise the risk of ventilator-induced lung injury [6]. Clinical research and comparative studies have been conducted to investigate the safety and effectiveness of PCV versus VCV during laparoscopic surgery. While both techniques can be utilized effectively, these studies suggest that PCV may provide better regulation of oxygenation and airway pressures, especially in individuals with impaired respiratory mechanics. However, the anesthesia team's experience, surgical circumstances, and patient-specific characteristics should all be taken into account when selecting a ventilation strategy [7].

The decision to choose between VCV and PCV during laparoscopic surgery is impacted by developments in anesthetic techniques and ventilatory technologies, in addition to physiological and clinical factors. Anesthesiologists can maintain ideal ventilation parameters and promptly address any deviations with the aid of advanced monitoring tools and alarms found on modern ventilators. Improved monitoring features, like real-time respiratory mechanics analysis, make it possible to more effectively customize ventilation plans to meet the needs of specific patients [8]. Additionally, to improve patient outcomes and safety, the chosen ventilation mode can be complemented by the incorporation of multimodal anesthesia approaches, such as the use of muscle relaxants and customized intraoperative fluid management. To improve these tactics and maintain the highest standards of care in the field, ongoing research and clinical innovation are crucial [9].

Objective of the review 

The purpose of this review is to assess and compare the effectiveness, safety, and clinical results of PCV versus VCV in laparoscopic surgery. It is aimed at examining the physiological effects of each mode of ventilation on respiratory mechanics and hemodynamics under increased intra-abdominal pressures, evaluating advantages and disadvantages in terms of oxygenation levels and airway pressures, and investigating recent clinical trials for evidence-based recommendations. Furthermore, it will explore new ventilatory technologies with multimodal anesthesia techniques to improve patient-tailored ventilatory strategies in order to promote optimal surgical outcomes.

## Review

Methodology

Search Strategies

A comprehensive search strategy was deployed in the literature review, involving multiple databases such as PubMed, Scopus, Web of Science, and Google Scholar. This involved “Pressure Control Ventilation,” “Volume Control Ventilation,” “laparoscopic surgery,” “respiratory mechanics,” “intra-abdominal pressure,” “oxygenation,” “airway pressures,” and "ventilator-induced lung injury" as the keywords used. Boolean operators were used to combine search terms and refine search results. Only articles published in English from January 2000 to June 2024 were included in this search to capture recent and relevant studies.

Criteria for Inclusion and Exclusion

Inclusion criteria were set up to identify studies that specifically compared PCV with VCV in laparoscopic surgery; had quantitative data on respiratory mechanics, hemodynamics, and patient outcomes; and were peer-reviewed. The exclusion criteria included nonlaparoscopic surgical procedures, case reports, review articles, editorials, and studies without full text.

Extraction of Data and its Synthesis

A total of 184 articles were initially identified using the search strategy. After removing duplicates, there were 137 articles. These papers were then screened based on titles and abstracts, giving a total of 56 documents for full-text review. Among these many papers that underwent thorough assessment against several inclusion/exclusion criteria, only eight meet this standard; hence, they have been considered for detailed analysis. The data extracted from these studies consisted of study design, sample size, patient demographics, ventilation parameters, intraoperative and postoperative outcomes, and major findings contained in the articles themselves. Table 1 includes a description of the articles included in the study.

**Table 1:** Description of studies included in the review PIP: Peak inspiratory pressure; VCV: volume-controlled ventilation; PCV-VG: pressure-controlled ventilation volume guarantee; P peak: peak airway pressure; P mean: mean inspiratory pressure; P plateau: plateau inspiratory pressure; Pao2: partial arterial pressure of oxygen; OLV: one lung ventilation; VATS: video-assisted thoracic surgery; PC: pressure control; VC: volume control

Sr no.	Author and year of publication	Participants	Outcome measures	Conclusion
1	Arjyal et al. (2022) [10]	A total of 100 patients who underwent laparoscopic surgery were undertaken for this study. The 50 in each group (PCV and VCV) were allotted randomly.	Peak airway pressure: mean airway pressure and lung compliance at different points of pneumoperitoneum	Respiratory mechanics improve when the patient is on pressure control ventilation compared to the volume control method of ventilation. However, there is no difference in hemodynamic parameters between the two methods
2	Wang et al. (2021) [11]	Eighty participants scheduled for laparoscopic surgery were enrolled in this prospective, randomized clinical trial	The absolute difference in PIP between VCV and PCV-VG during the first hour of pneumoperitoneum. Changes in PIP from five minutes after induction of anesthesia to 60 minutes after pneumoperitoneum were also evaluated as part of the study's primary outcomes. Hemodynamic variables were measured as secondary endpoints in the research, focusing on parameters such as blood pressure, heart rate, and cardiac output	Elderly patients undergoing laparoscopic surgery with a laryngeal mask airway concluded that PCV-VG was superior to VCV in providing ventilation with lower PIP and greater dynamic compliance
3	Mihalj et al. (2017) [12]	This study included 60 patients aged between 18 and 70 years of age	Respiratory and hemodynamic parameters	In the patient groups under observation, both PCV and volume-controlled ventilation proved to be equally successful in preserving appropriate ventilation, oxygenation, and hemodynamic stability. However, a comparison of obese patients showed some benefits of PCV that need further study, given the current rate of change
4	Wang (2015) [13]	Eight randomized controlled trials with a total of 428 participants	Respiratory mechanics (including peak airway pressure, plateau pressure, mean airway pressure, compliance, airway resistance, minute volume, end-tidal CO2 tension and tidal volume) and hemodynamic parameters (including heart rate and mean arterial pressure)	According to a meta-analysis, patients who had laparoscopic surgery with PCV and VCV had identical hemodynamic parameters; however, the respiratory data for PCV patients was slightly better
5	Dion et al. (2014) [14]	This was a prospective cross-over cohort trial (n = 20). In random sequence, each patient received the three modes of ventilation for 20 minutes during the laparoscopic portion of the procedure	PIP, exhaled tidal volume, respiratory rate, and oxygen saturation were recorded every five minutes. At the end of 20 minutes, an arterial blood gas was obtained	In adolescents and young adults undergoing laparoscopic bariatric surgery, PCV-VG and PC were superior to VC ventilation in their ability to provide ventilation with the lowest PIP
6	Pu et al. (2014) [15]	Twenty participants were recruited and equally assigned into two groups in a controlled, randomized, cross-over design	Blood gas analysis, P peak, P mean, and P plateau were measured at four different time points: (1) 30 min after total lung ventilation (TLV); (2) 30 min after one-lung ventilation (VCV or PCV-VG); (3) 30 min after shifting to the other ventilatory mode and (4) 30 min after reconstruction of TLV	When it came to offering the lowest PIP for ventilation during laparoscopic bariatric surgery, PCV-VG and PC outperformed VC ventilation in the case of adolescents and young adults
7	Sinha et al. (2010) [16]	Two groups of 20 each to receive either PCV or VCV	Assessment of PIP differences between VCV and PCV-VG during the first hour of pneumoperitoneum. Evaluation of changes in PIP from five minutes after anesthesia induction to 60 minutes after pneumoperitoneum to compare ventilation strategies	PCV should be the preferred mode to provide PPV when using the size-1 cLMA in babies weighing 2.5–5 kg, in view of less gastric insufflation associated with it for surgeries of brief duration
8	Wang et al. (2021) [17]	The infants were divided into two groups according to the One lung ventilation pattern: group G (n = 30, receiving PCV-VG) and group V (n = 28, receiving VCV)	Mean arterial pressure, heart rate, P peak, P mean, dynamic compliance, PaO2	Mechanical ventilation using the PCV-VG mode is possible in infants when performing OLV during VATS. Compared to VCV, PCV-VG can offer lower P peak and P mean, improve lung compliance, and achieve better oxygenation

This review compares the safety and effectiveness of VCV with PCV during laparoscopic surgery, emphasizing the differences in their effects on patient outcomes and respiratory mechanics. Intra-abdominal pressure is naturally raised during laparoscopic surgeries, and this might have a substantial impact on hemodynamic and respiratory dynamics. Research suggests that by preserving lower peak airway pressures and enhancing oxygenation, PCV can be beneficial in these situations. A study discovered that PCV, as opposed to VCV, increased oxygenation during laparoscopic obesity surgery. Similarly, PCV improved respiratory mechanics control during long-term anesthesia. However, VCV guarantees a constant tidal volume with each breath, which can be essential for preserving accurate ventilation control, especially in individuals with varying lung compliance.

On the other hand, this mode may result in larger peak airway pressures, raising the possibility of lung damage brought on by a ventilator. In the context of ventilation modes, particularly PCV and VCV, studies have shown varying effects on pCO_2_ levels. While a study comparing PCV and VCV in infants and young children undergoing spinal cord detethering surgery did not find significant differences in pCO_2_ levels [18], another study in an animal model found that PCV and VCV at similar tidal volumes did not result in different pCO_2_ levels [19]. Additionally, research on neonatal ventilation modes indicated that volume-targeted ventilation (VTV), compared to pressure-limited ventilation (PLV), led to more stable pCO_2_ levels, reducing the risk of hypocarbia associated with lung damage [20]. Furthermore, a meta-analysis in adults undergoing one-lung ventilation (OLV) revealed no significant differences in pCO_2_ levels between PCV and VCV, suggesting comparable effects on carbon dioxide elimination [21].

Discussion 

The decision to use PCV or VCV during laparoscopic surgery depends on the raised intra-abdominal pressure in these procedures, which significantly affects respiratory and circulatory physiology [22]. Arjyal et al. evaluated the use of PCV in 100 patients in the process of a laparoscopic study, and the result demonstrated that the use of PCV delivers improved lung mechanics than VCV; no significant changes were observed in the hemodynamic parameters, meaning that more beneficial in lung mechanics without compromising more hemodynamic stability. Similarly, Wang et al. performed a prospective randomized clinical trial of 80 subjects to know the difference in the use of PCV-VG on the ventilation parameter VCV [13-26]. They postulated that PCV was advantageous in the ventilation of aged patients who had undergone laparoscopic surgery with a laryngeal mask airway in terms of lesser PIP and higher dynamic compliance. Mihalj et al. recruited 60 patients aged from 18 to 70 years old, where both PCV and VCV were comparable in keeping the correct ventilation, oxygenation, and hemodynamic state. Still, a slight advantage of PCV in obese patients needs further research [27-30].

Wang conducted a meta-analysis of eight randomized controlled trials with 428 participants, revealing that PCV and VCV had identical hemodynamic parameters, with slightly better respiratory data for PCV patients. Dion et al. conducted a prospective cross-over cohort trial with 20 adolescents and young adults undergoing laparoscopic bariatric surgery, finding that PCV and PC were superior to VC in providing ventilation with the lowest PIP. Pu et al. conducted a controlled, randomized, cross-over study with 20 participants, demonstrating that PCV-VG and PC outperformed VC in offering the lowest PIP during laparoscopic bariatric surgery [11,17]. Sinha et al. studied two groups of 20 infants and concluded that PCV should be preferred for providing positive pressure ventilation with less gastric insufflation during brief surgeries. Finally, Wang et al. conducted a study on infants undergoing OLV during video-assisted thoracoscopic surgery (VATS), finding that PCV-VG offered lower peak airway pressure (P peak) and mean inspiratory pressure (P mean), improved lung compliance, and better oxygenation compared to VCV [31].

Given these findings, PCV can maintain lower peak airway pressures, potentially reducing the risk of volutrauma and barotrauma, making it a preferred mode in many situations. However, VCV guarantees a constant tidal volume with each breath, which is essential for maintaining precise ventilation control, especially in patients with varying lung compliance, despite potentially higher peak airway pressures that may increase the risk of ventilator-induced lung damage. Ultimately, the choice between PCV and VCV should be individualized, considering the anesthesia team's experience, the type of surgery, and patient-specific factors. Advanced ventilatory technology and real-time monitoring systems can aid in optimizing ventilation strategies, ensuring responsiveness to dynamic changes in respiratory mechanics during laparoscopic procedures. Integrating multimodal anesthesia approaches, such as the use of muscle relaxants and tailored intraoperative fluid management, can further improve patient outcomes. Ongoing clinical research and innovation are necessary to refine these strategies and ensure optimal care in the complex field of laparoscopic surgery [32-35].

## Conclusions

A comparison between VCV and PCV during laparoscopic surgery indicates specific benefits and possible drawbacks for each method. Given the elevated intra-abdominal pressures during these surgeries, PCV typically offers superior regulation of oxygenation and airway pressures. Conversely, because of its higher peak airway pressure, VCV increases the risk of ventilator-induced lung injury while maintaining accurate ventilation control and a constant tidal volume. To maximize the results of laparoscopic procedures, the ventilation strategy should be customized based on the patient's unique respiratory mechanics and the surgical setting, utilizing cutting-edge ventilatory technologies and real-time monitoring.
